# Chemogenetic tools for modulation of spatial learning in dopamine transporter deficient rats

**DOI:** 10.3389/fnins.2025.1615208

**Published:** 2025-07-21

**Authors:** Arina A. Gromova, Taisiia S. Shemiakova, Mikhail G. Khotin, Evgeniia N. Petrunina, Anastasia D. Belskaya, Raul R. Gainetdinov, Natalia P. Kurzina, Anna B. Volnova

**Affiliations:** ^1^Institute of Translational Biomedicine, Saint Petersburg State University, Saint Petersburg, Russia; ^2^Biological Faculty, Saint Petersburg State University, Saint Petersburg, Russia; ^3^Institute of Cytology, Russian Academy of Sciences, St. Petersburg, Russia; ^4^Saint Petersburg University Hospital, Saint Petersburg, Russia

**Keywords:** hyperdopaminergia, dopamine transporter, knockout rats, chemogenetic, learning, Hebb-Williams maze

## Abstract

**Objectives:**

We used the chemogenetic tools for the activation of norepinephrine (NE) release in the prefrontal cortex (PFC) of dopamine transporter knockout (DAT-KO) rats. The objective of this study was to evaluate the effect of chemogenetic activation of NE release in the PFC on the performance of a spatial behavior task by DAT-KO rats. The rats DAT-KO rats with deletion of DAT gene were created as a valuable model for persistently elevated extracellular DA levels. The DAT-KO rats show marked behavioral abnormalities: impulsivity, stereotypy and reduced learning ability. Such hyperdopaminergia is thought to be one of the causes of disorders such as schizophrenia, mania and attention deficit hyperactivity disorder (ADHD). The Locus Coeruleus (LC) is a critical area in the brain that plays an important role in control of several physiological and behavioral processes due to the existence of extensive connections to cortical and subcortical structures. Its activity can modulate both norepinephrine and dopamine neurotransmission, particularly in the PFC.

**Materials and methods:**

We used canine adenovirus type 2 (CAV2) to selectively activate LC-NA neurons in DAT-KO rats. The chemogenetic modulation of spatial learning in knockout and wild-type (WT) rats was tested in the Hebb-Williams maze. Variables such as the distance traveled, time taken to reach the goal box, number of errors and the perseverative patterns of activity were analyzed.

**Results:**

Norepinephrine release from the LC to the PFC reduced hyperactive behavioral patterns in rats lacking the dopamine transporter (DAT-KO rats) with spontaneously elevated dopamine transmission. These manipulations in hyperdopaminergic mutants also caused amelioration of cognitive abnormalities in spatial learning task by decrease the perseverative activity and the number of visits to the error zones. Furthermore, chemogenetic activation of NE neurotransmission in these animals significantly improved their performance.

**Conclusion:**

The results obtained in this study highlight an important modulatory role of NE transmission from LC to PFC on hyperactivity and cognitive dysfunctions of hyperdopaminergic DAT-KO rats lacking the dopamine transporter.

## Introduction

The brainstem nucleus Locus Coeruleus (LC) is involved in wide range of functions, including sensory processing, motor behavior, and cognition (Bari et al., 2020; Poe et al., 2020). It contains one of the largest populations of norepinephrine (NE) neurons from different brain areas (Sara, 2009). The LC noradrenergic system provides widespread innervation to many brain structures of the CNS and affect cognitive processes in several key regions (Radley et al., 2008). It has been shown that LC activity is low during routine behaviors such as grooming or feeding, whereas its neurons respond with a phasic burst of activity to stimuli in all sensory modalities when they are novel. This type of activity leads to behavioral adaptation to the new context (Berridge and Waterhouse, 2003; Bouret and Sara, 2005). The reciprocal relationship between LC and prefrontal cortex (PFC) is thought to support this behavioral reorganization (Agster et al., 2013; Bouras et al., 2023).

Catecholamines play an important role in human and animal behavior. Their wide distribution in the brain areas provides for a vast functional diversity. Studies of the interactions between the dopamine (DA) and norepinephrine systems in the CNS suggest that these systems may act in an overlapping and parallel manner (Ranjbar-Slamloo and Fazlali, 2020). The correct balance of catecholamines in the brain is important for the proper organization of many types of behavior. The absence of this balance can lead to the development of various psychiatric disorders, including attention deficit hyperactivity disorder (ADHD) (Ko et al., 2013; Natsheh and Shiflett, 2018; Russell, 2011; Tripp and Wickens, 2012).

It is believed that ADHD development mainly arise due to the dysfunction of the DA system (Ng et al., 2014; Viggiano et al., 2004). Some cases of ADHD are linked to DNA damage in genes encoding protein transporters of dopamine (DAT) and norepinephrine (NET), which are located in the synaptic membrane and ensure the reuptake of released molecules for their next use (Blum et al., 2008). It was proposed that abnormalities in the DA and the NE systems may play the key role in ADHD development (Del Campo et al., 2011; Nikolaus et al., 2010).

The rats with deletion of DAT gene (DAT-KO rats) were created as a valuable model for ADHD with emphasis on various aspects of DA system dysfunctions (Leo et al., 2018; Savchenko et al., 2023; Sukhanov et al., 2019; Vengeliene et al., 2017). Rats lacking SLC6A3, the DAT coding gene, were generated using zinc finger nucleases (ZFN) technology. DAT-KO rats are an animal model of persistently elevated extracellular DA levels (Leo et al., 2018). The knockout rats show marked behavioral abnormalities: impulsivity, stereotypy and reduced learning ability (Belskaya et al., 2024; Kurzina et al., 2020, 2022; Sukhanov et al., 2019). Such hyperdopaminergia is thought to be one of the causes of disorders such as schizophrenia, mania and ADHD (Savchenko et al., 2023).

The PFC plays an important role in working memory (Vogel et al., 2022) and is innervated by NE pathways arising from the locus coeruleus and DA pathways arising from the ventral tegmental area (Xing et al., 2016). Interactions between NE and DA in the PFC and striatum are crucial for the organization of complex behaviors, including spatial learning (Horst and Laubach, 2009, 2012). In mice, enhancing the activity of the pathway from the PFC to the dorsal striatum alleviated working memory impairment in the maze (Wilhelm et al., 2023). We think that revealing interactions of these two systems in the cortex of DAT knockout animals may help to understand mechanisms of different pathological states and create the new approaches to their treatment.

Chemogenetic tools have been widely used to explore brain function and connections between brain regions (Campbell and Marchant, 2018; Roth, 2016; Smith et al., 2021). This method relies on cell-specific viral delivery to express designer receptors that are exclusively activated by designer drugs (DREADD). This tool can activate specific neuronal pathways by applying the specific molecular ligand. Now, chemogenetic modulation of LC activity has been used to study sensorimotor integration and selective modulation of LC-PFC functional connectivity (Kabanova et al., 2024a, 2024b).

In this study, we evaluated the effect of activation of NE release in the PFC on the performance of a spatial behavior task in DAT-KO rats. An increase in NE levels was achieved by chemogenetic modulation of LC neuronal activity using the viral vector CAV-2. We used canine adenovirus type 2 (CAV2) – the viral vector carrying the noradrenergic cell-specific promoter, activated DREADDs (Designer Receptor Exclusively Activated by Designer Drugs) to selectively activate LC-NA neurons in DAT-KO and wildtype (WT) rats. CAV2 (CAV-PRS-hM3Dq-mCherry) was injected into the PFC to retrogradely transduce LC neurons projecting to the PFC. After transduction, the neurons acquire DREADDs, and an increase in NE was achieved by chemogenetic modulation of LC neuronal activity using the specific ligand clozapine (Clz). After activation of LC NE neurons, the impact of NE enhancing on spatial task learning in the Hebb-Williams maze by DAT-KO and WT rats was investigated. We suggest that chemogenetic modulation causing an increase of NE levels in the PFC may alleviate hyperactivity and cognitive deficits of hyperdopaminergic DAT-KO rats.

## Materials and methods

### Animals

Thirty two DAT-KO and thirty two WT littermate rats, males and females of the age 4 months, were used in the experiments. Our observations have shown that there are no significant differences in behavior between the sexes. Therefore, due to the limited number of DAT-KO males, 3 female rats of both genotypes were added to each group. To analyze the effects of chemogenetic modulation of LC neurons, a group of DREADD-expressing animals (8 DAT-KO and 8 WT) and a control group of rats that did not receive CAV2 microinjection (8 DAT-KO and 8 WT) were formed.

All experimental procedures were conducted in compliance with requirements regarding the care and treatment of laboratory animals and the Ethics committee of Saint Petersburg State University, St. Petersburg, Russia (protocol No. 131-03-10 of 22 November 2021). Before the experiments, rats were maintained in IVC cages (RAIR IsoSystem World Cage 500; Lab Products, Inc.) with free access to food (BioPro, Russia) and water, at a temperature of 22 ± 1 degrees C, 50–70% relative humidity and a 12 h light/dark cycle (light from 9 am). Experiments were carried out between 1 pm and 5 pm.

The use of mixed groups containing both male and female rats was the limitation of our research. Most behavioral studies are conducted on male rats and female rats are often overlooked. However, the inclusion of females in experiments is important both for understanding the mechanisms underlying the observed phenomena and for analyzing the effects of pharmacological agents in translational studies. There is data suggesting that spontaneous exploration, anxiety-like behavior, working memory, and long-term memory for objects were similar in middle-aged male and female rats (Colettis et al., 2022). Additionally, recent studies comparing the behavior of male and female rats revealed no significant differences in behavioral tests involving positive motivation (Pupikina and Sitnikova, 2023). Studies conducted on DAT-KO rats confirmed that data obtained from behavioral experiments involving male and female rats is comparable (Illiano et al., 2021).

### Viral vector microinjection

For transduction of LC noradrenergic neurons, microinjection of viral vector into PFC was performed. Microinjection of viral vectors into the PFC was used for transduction of LC NE neurons. The CAV-2-carrying DREADD coupled to the mCherry sequence (CAV PRS hM3D(Gq)-mCherry, 2.5 × 10^12^ particles/ml; PVM, Montpellier) was used. To ensure the vitality of virus particles they were stored at −80°C and were defrosted only immediately before injection. Eight DAT-KO and eight WT rats were anesthetized with Isoflurane, 1% in 100% oxygen (Chemical Iberica Produktos Veterinarios, Croatia). CAV PRS hM3D(Gq)-mCherry was injected 600 nL each into the left and right PFC. Microinjection coordinates were AP: +3.0 mm, ML: ± 0.8 mm. The virus dose was divided into three depth points: −3.6, −3.4, −3.2. The vector-containing substance was delivered using a microsyringe pump (UMC4 MicroSyringe Pump Controller, World Precision Instruments) at 150 nL/min. A syringe (Hamilton 701 RN Syringe, 10mcl) with a glass microcapillary nozzle made on a puller (Narishige PC-100) was placed in the pump. The glass microcapillary was lowered to −3.6 and then raised to the next injection point, pumping in 200 nL at a time.

After surgery, the animals were given the necessary post-operative care. Over the next 6 weeks, the rats recovered and the viral vector retrogradely transduced via NE neurons into the LC. The control groups of rats (8 DAT-KO and 8 WT) did not receive virus microinjection.

### Hebb-Williams maze apparatus and experimental setup

**Figure 1:**
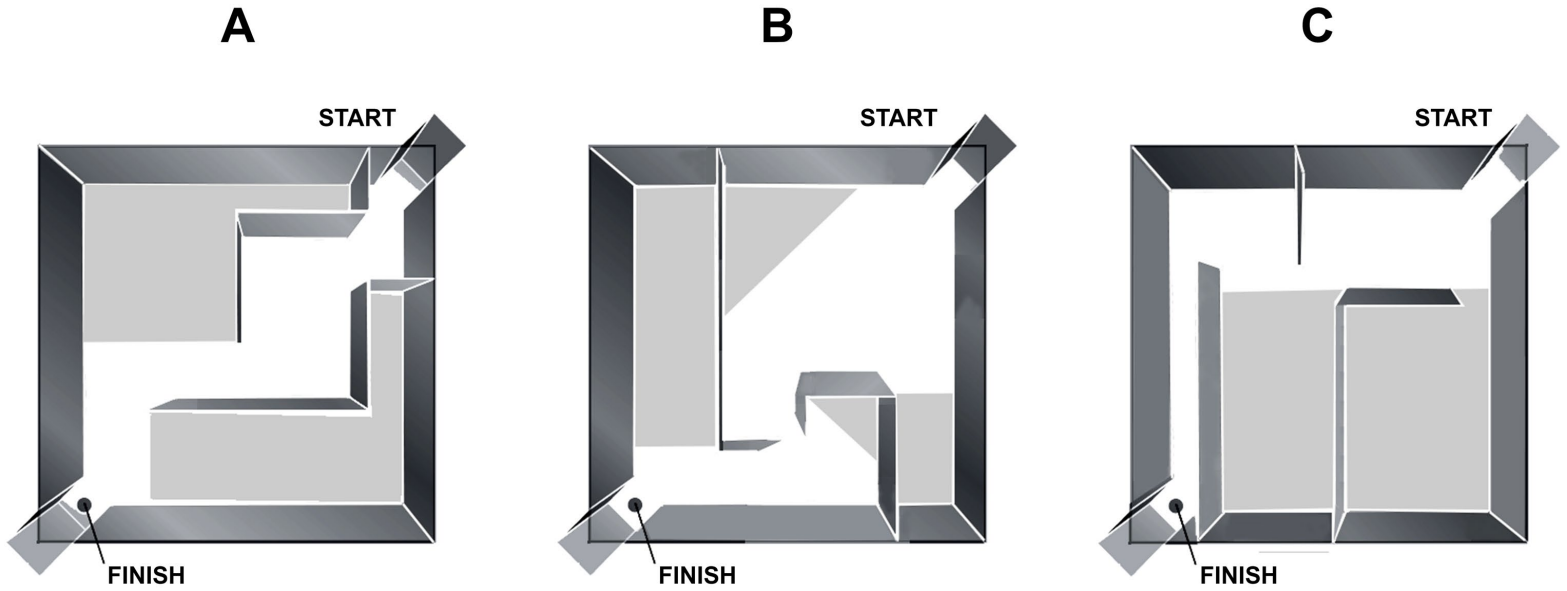
Scheme of the Hebb-William maze and its inner wall configurations, used for learning **(A)** and drug testing **(B,C)**. The location of the start and finish chambers are marked in the opposite corners. Food reinforcement is indicated by a circle.

The Hebb-Williams maze, which consists of a set of internal walls to create different maze configurations within an enclosed arena, was chosen for behavioral testing (Pritchett and Mulder, 2004). The arena represents a 75 × 75 cm square platform surrounded by 25 cm walls. The start and finish chambers were located at opposite corners. The inner walls can be placed in different configurations to create the correct path as well as the error zones. Animals need to learn to find the way through the maze from start to finish to obtain a food reward (Figure 1). For 5 days prior to training and throughout the experiment, the rats were given food at 90% of their normal diet to create food motivation. Popcorn loops (Nestlé, S. A., weight 0.2 g) were used as a reward. Each animal was weighed daily before and throughout the experiment. Animals were handled in the experimental room to habituate them to experiment conditions.

The behavior of two groups of rats was accessed: rats expressing DREADD (8 KO + 8 WT) and control group of rats without DREADD (8 KO + 8 WT). For female rats (3 females in each group), the estrous cycle was monitored, and behavioral testing was not performed on days of the proestrus phase.

On the first day animals were placed into the arena without inner walls to familiarize them with the setup. Then rats were trained in the “learning” arena configuration for 3 days (Figure 1A). Each day animals were given three trials to complete the task. On the fourth experimental day, the arena configuration was changed, and the animals began receiving intraperitoneal injections 30 min before the experimental session. In the new maze configuration (Figure 1B), rats were trained after vehicle i.p. injection. After 3 days, the animals were tested in the following maze configuration (Figure 1C) after i.p. injection of Clz (1 mg/kg in 0.0001 M HCL solution).

Video was acquired from the camera mounted above the maze and the behavioral variables were analyzed with software EthoVision XT11.5 (Noldus Information Technology, Leesburg, VA, United States). Following characteristics were chosen to analyze: distance traveled, time spent on task completion, number of errors, and return runs.

### Immunohistochemistry

Neurons transduced by a viral vector had a DREADD receptor on their membrane coupled to mCherry, a fluorescent reporter protein. At the end of the experiment, we verified accuracy of virus injections and evaluated the number of neurons transduction in LC. Animals were deeply anesthetized with a mix of 200 mg/kg Zoletil with 16 mg/kg Xylazine, then transcardially perfused with 0.9% NaCl (100 mL) followed by 4% paraformaldehyde (PFA, 100 mL) in 0.1 M PBS (pH 7.4). Extracted brain tissue was placed for 24 h in PFA for post-fixation at room temperature (RT). The brains were placed in increasing concentration sucrose solutions for cryoprotection. Then, 50 μm frontal free-floating sections of LC were prepared on a cryostat Leica CM-3050S. The sections were stored in 0.1% NaN3 at +4°C.

To assess the number of NE neurons transduced by viral vector we performed the procedure of double immunostaining. After being washed in PBS, the sections were processed in citrate buffer (pH 6.0) for antigen retrieval and blocked with 5% Normal Goat Blocking Buffer (Elabscience, E-IR-R111) for 1.5 h at RT. Then, the sections were incubated in the primary antibodies: mouse anti-DβH antibody (1:2000, MAB308, Chemicon) and rabbit anti-mCherry antibody (1:1000 Cat#632496, Takara Bio, United States) (Falcy et al., 2020) for 24 h at RT, and the secondary antibody: donkey anti-mouse IgG Alexa Fluor 488 (1:500, abcam, ab150105, UK) and CyTM3 AffiniPure donkey anti-rabbit IgG (1:800, AB_2307443, Jackson ImmunoResearch Labs, United States) for 2 h at 37°C. Finally, the sections were mounted in aqueous medium Fluoroshield with DAPI (Sigma-Aldrich, Cat # F6057, United States).

Stained sections were imaged on fluorescence microscope (Leica DMI6000 objective 10x) with a buildin 8MP CCD color digital camera. mCherry- and DβH-positive neurons were counted manually with Fiji ImageJ software (Schindelin et al., 2012). To evaluate the scale in which virus had transduced LC neurons, the percentage of mCherry+ neurons to DβH + neurons was calculated.

### Statistical analysis

All values were averaged over all trials for 3 days per animal, and then groups of rats were compared. The recorded parameters were averaged over all trials for 3 days for each animal and then the groups of rats were compared. The normality of the distribution was preliminarily assessed using the Kolmogorov–Smirnov test. We used a two-way analysis of variance (ANOVA), analyzing the genotype factor (DAT-KO or WT) and the treatment factor (saline of Clz administration) with Fisher’s LSD post-test for groups comparison. The *t*-test or the non-parametric Mann–Whitney test was used for the comparison of the mean values. All calculations were performed in GraphPad Prism 8 (GraphPad Software, Inc., San Diego, CA, United States).

## Results

### Hebb-Williams maze

The rats in the experimental group received the microinjection of the virus carried artificial receptors (DREADD+ group) on the membrane of LC neurons that were only activated by the specific ligand Clz. The i.p. injection of vehicle did not affect their activity. Therefore, we compared the behavioral parameters of rats in the Hebb-Williams maze after i.p. injection of vehicle or Clz (DREADD activator). A control group of animals did not receive CAV2 microinjection (DREADD- group). In this group of rats, we also compared the behavioral parameters of the rats after i.p. injection of vehicle or Clz to verify that the differences found were not due to the effect of Clz per se.

**Figure 2:**
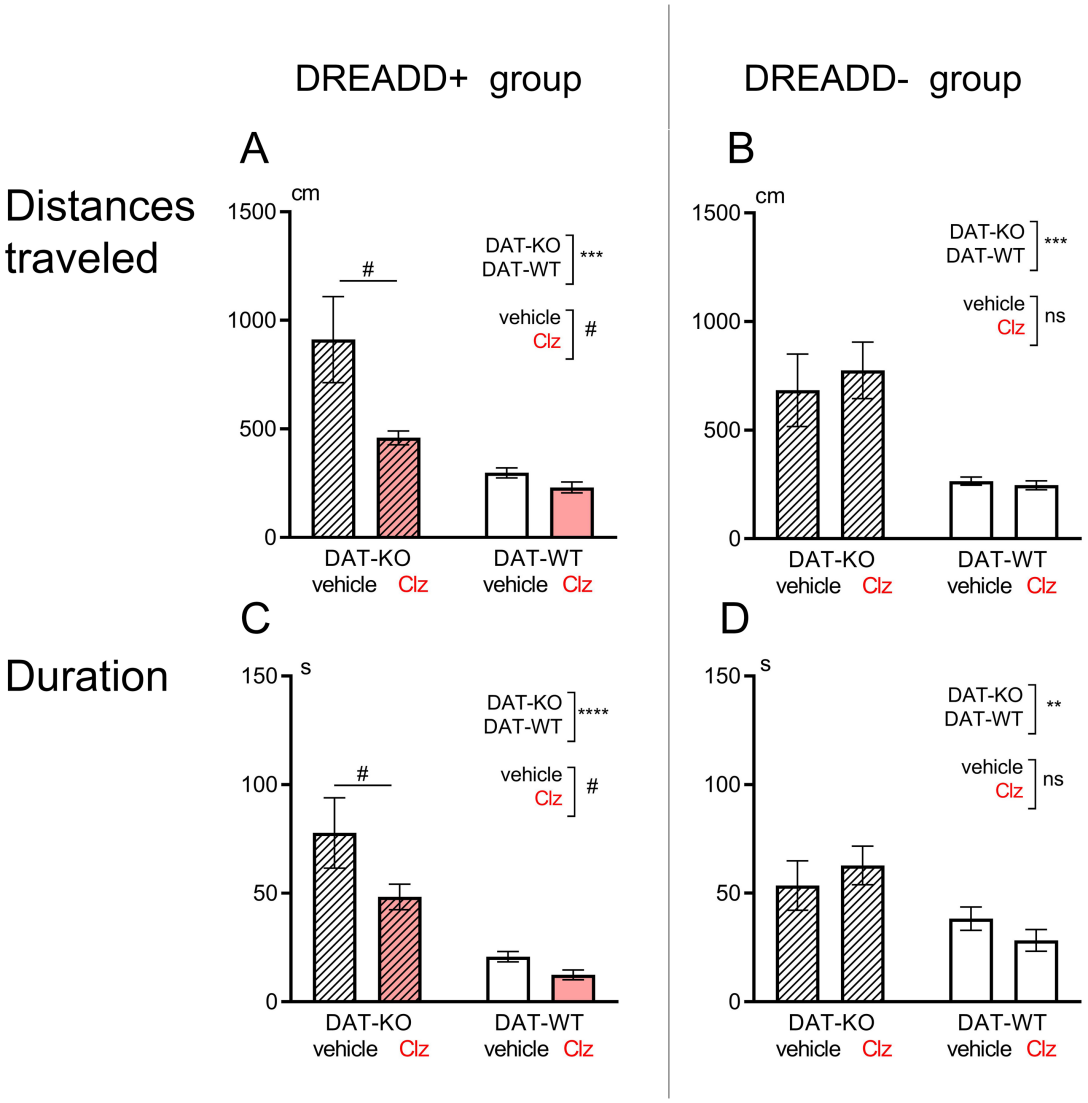
Comparison of distances traveled **(A,B)** and duration of test performance (time spent, **C,D**) in Hebb-Williams maze in DAT-KO and WT rats in DREADD+ (left side, **A,C**) and DREADD- (right side, **B,D**) groups. Results are presented as the mean ± SEM; **p < 0.01; ***p < 0.001; ****p < 0.0001; two-way ANOVA, analyzing the genotype factor (DAT-KO or WT); ^#^*p* < 0.05; analyzing the treatment factor (vehicle of Clz administration); ns, *p* > 0.05.

The hyperactive behavior of DAT-KO rats has been shown in a large number of publications (Adinolfi et al., 2019; Kurzina et al., 2020, 2021, 2022; Leo et al., 2018; Savchenko et al., 2022; Volnova et al., 2023). A comparison of the behavior of DAT-KO and WT rats in the present study also supports this observation. The results of the experiments showed that the distance traveled by DAT-KO rats was significantly greater than in WT rats (911.6 ± 198.1 in DAT-KO versus 297.7 ± 23.4; *p* < 0.001, Mann Whitney test, Figures 2A,B). The knockout rats also spent significantly more time performing the test (77.7 ± 16.2 versus 20.8 ± 2.4; *p* < 0.001, Mann Whitney test, Figures 2C,D). This fact indicates a pronounced hyperlocomotion in DAT-KO rats compared to WT animals.

For the DREADD+ group of DAT-KO rats, two-way ANOVA analysis of the “distance” parameter shows a significant difference: on the genotype factor, *F* (1, 27) = 16.07, *p* = 0.0004; on the treatment factor (vehicle-Clz), *F* (1, 27) = 6.12, *p* = 0.0199; Figure 2A. Similarly, i.p. administration of Clz in DAT-KO + rats resulted in a significant reduction in duration of test performance [two-way ANOVA analysis, treatment factor (vehicle-Clz), *F* (1, 27) = 4.46, *p* = 0.044; Figure 2C]. Thus, activation of LC noradrenergic neurons in the DREADD+ group of rats by Clz resulted in a significant decrease in the distance traveled and the time spent in Hebb-Williams maze by DAT-KO rats. In WT rats, i.p. administration of Clz and activation of DREADD caused no change.

In the DREADD- groups of rats, i.p. Clz administration did not cause any significant changes in the distance traveled by the animals: two-way ANOVA analysis, treatment factor (vehicle-Clz) [*F* (1, 28) = 0.118, *p* = 0.734; Figure 2B] and in the time needed to complete the test [two-way ANOVA analysis, treatment factor (vehicle-Clz), *F* (1, 28) = 0.0017, *p* = 0.968; Figure 2D]. We can conclude that i.p. administration of Clz alone at the chosen dose (1 mg/kg) did not cause any significant changes in the parameters “distance” and “test performance time” in either knockout rats (two-way ANOVA, Fisher’s LSD test, *p* = 0.546 and *p* = 0.426 respectively) or WT animals (two-way ANOVA, Fisher’s LSD test, *p* = 0.902 and *p* = 0.394 respectively).

**Figure 3:**
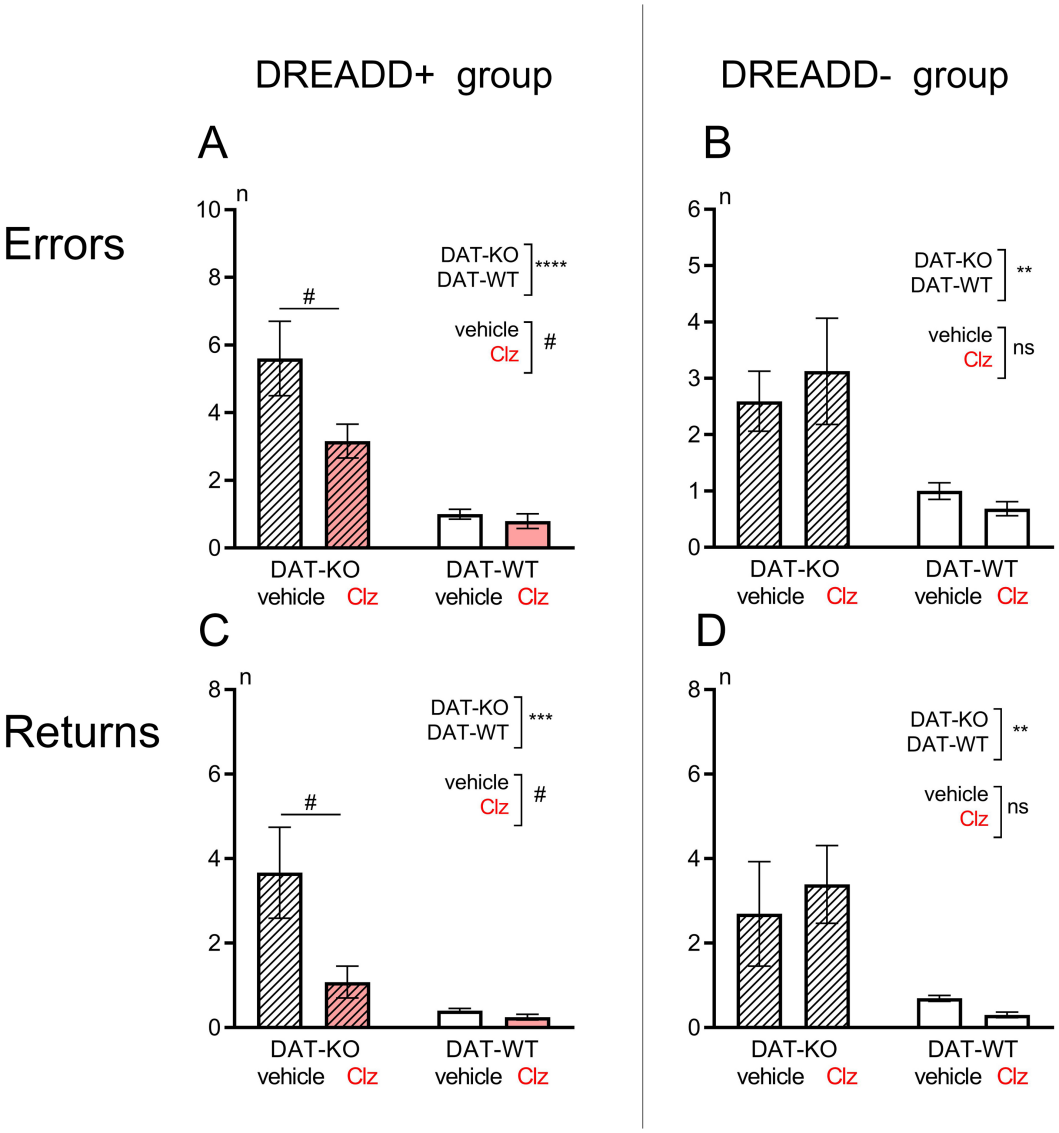
Comparison of the number of error zone visits **(A,B)** and the number of the return runs (time spent, **C,D**) in Hebb-Williams maze in DAT-KO and WT rats in DREADD+ (left side, **A,C**) and DREADD- (right side, **B,D**) groups. Results are presented as the mean ± SEM; **p < 0.01; ***p < 0.001; ****p < 0.0001; two-way ANOVA, analyzing the genotype factor (DAT-KO or WT); ^#^*p* < 0.05; analyzing the treatment factor (vehicle of Clz administration); ns, *p* > 0.05.

Previous studies of the behavior of DAT-KO rats in spatial orientation tests have convincingly shown that they are less successful in achieving a goal compared to WT rats (Kurzina et al., 2020, 2022; Volnova et al., 2023). The present study also confirmed these findings. The number of error zone visits was significantly higher in DAT-KO rats compared to WT animals (5.6 ± 1.1 in DAT-KO versus 1.0 ± 0.2; *p* < 0.001, Mann Whitney test, Figures 3A,B).

DAT-KO rats are also characterized by marked stereotypy and a tendency to perform inefficient repetitive motor acts (Belskaya et al., 2024). In the present experiment, we observed multiple returns to the start chamber of the maze in each experimental session without reaching the goal and without food reinforcement in DAT-KO rats. The level of such perseverative activity in DAT-KO rats was significantly higher than in WT rats (3.7 ± 1.1 in DAT-KO versus 0.4 ± 0.1; *p* < 0.001, Mann Whitney test, Figures 3C,D).

There was a significant decrease in the number of error zone visits (two-way ANOVA, the treatment factor, *F* (1, 27) = 4.45, *p* = 0.044; Figure 3A) and the number of the return runs [two-way ANOVA, the treatment factor, *F* (1, 27) = 5.51, *p* = 0.027; Figure 3C] in rats of the DREADD+ group of DAT-KO rats after clozapine injection. In the DREADD+ group of WT rats, i.p. administration of Clz and activation of DREADD caused no change: activation of DREADD did not alter the efficiency of test performance (number of error zone entries, Figure 3A) or the number of returns to the start chamber (Figure 3C).

In the DREADD- groups of rats, i.p. administration of Clz caused no changes in the number of rats’s visits the error zones [two-way ANOVA, the treatment factor, *F* (1, 28) = 0.040, *p* = 0.844; Figure 3B] or the number of return escapes [two-way ANOVA, the treatment factor, *F* (1, 28) = 0.039, *p* = 0.845; Figure 3D]. In both DAT-KO and WT rats, the i.p. administration of Clz did not alter the efficiency of the test performance (Figure 3B) or the level of perseverative reactions (Figure 3D). The administration of Clz alone did not cause any significant changes in the number of rats’s visits the error zones the number of return escapes in either knockout rats (two-way ANOVA, Fisher’s LSD test, *p* = 0.499 and *p* = 0.530 respectively) or WT animals (two-way ANOVA, Fisher’s LSD test, *p* = 0.691 and *p* = 0.725 respectively).

### Immunohistochemistry

**Figure 4:**
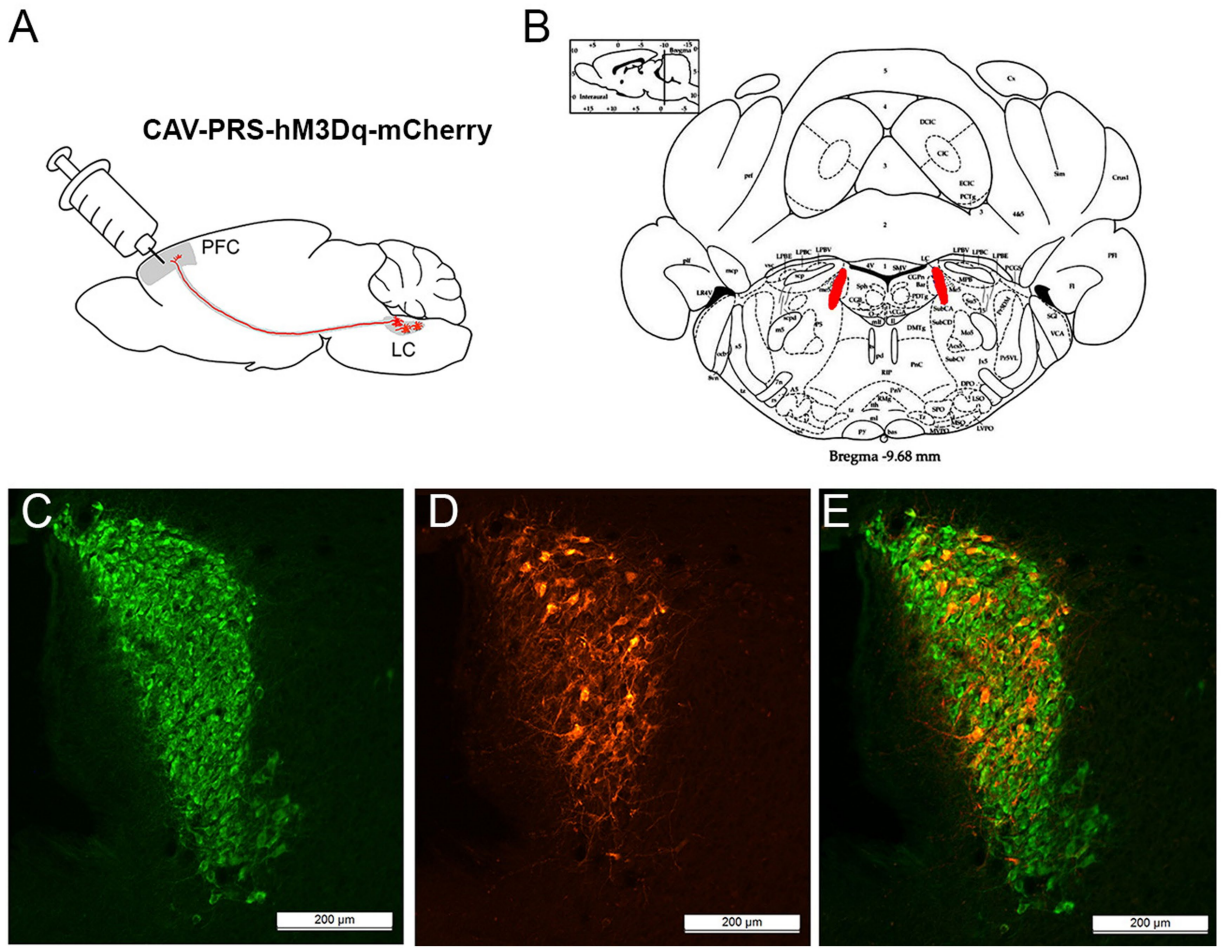
Immunofluorescence staining of rat brain LC neurons after administration of CAV PRS hM3D(Gq)-mCherry virus to the PFC **(A,B)**; green fluorescence is DβH-positive neurons **(C)**, red fluorescence is mCherry-positive neurons **(D)**, double-labeled neurons appear yellow-orange **(E)**.

An immunohistochemical morphological control of the viral vector transduction was performed at the end of the experiment (Figure 4). Together with the DREADD expression cassette, the fluorescent marker mCherry was delivered to the PFC of rats in the experimental DREADD+ group (Figure 4A). This allowed to visualize the transducted neurons. The degree of transduction was assessed by double immunofluorescence staining using antibodies against dopamine beta-hydroxylase (DβH) (Figure 4C) and mCherry (Figure 4D).

It was found that in WT rats 24.5 ± 1.7% of all DβH-positive neurons were transduced with the CAV2 PRS hM3D(Gq)-mCherry viral vector, whereas in DAT-KO rats the transduction rate was only 8.7 ± 1.2%; significance of differences by Manni-Whitney test *p* < 0.0001. Thus, we have shown that microinjection of CAV2 into the rat PFC results in efficient retrograde penetration of the viral vector into LC neurons and stable transduction of the NE neurons of this brain structure. In analyzing the data, it should be taken into account that in the LC of DAT-KO rats there are significantly fewer neurons carrying the artificial DREADD receptor, which may reduce the efficiency of chemogenetic activation of these neurons.

## Discussion

The Locus Coeruleus is the main source of NE in the PFC (Benarroch, 2017). LC is described as very important for implementing many behavioral functions such as arousal, attention and spatial memory (Breton-Provencher et al., 2021). Using chemogenetic and optogenetic methods, it has been shown that the LC is involved in the modulation of wakefulness (Carter et al., 2010; Naganuma et al., 2021), cognitive function (Uematsu et al., 2017) and stress-related behavior (Hirschberg et al., 2017; McCall et al., 2017).

The coexistence of DA and NE terminals in the PFC has been described previously (Robbins and Arnsten, 2009). It is proposed that their interactions may play a key role in the realization of complex behavior, and a lack of balance between DA and NE may lead to the development of pathophysiological processes, including ADHD symptoms (Howes et al., 2017; Winograd-Gurvich et al., 2006). The overlapping functions of the neuromodulators can provide a new approach to the mechanisms of neuropsychiatric disorders such as depression (Nutt, 2008), schizophrenia (Howes et al., 2017; Winograd-Gurvich et al., 2006), and ADHD (Kessi et al., 2022; Koirala et al., 2024).

According to the dopamine hypothesis of schizophrenia, increased dopamine (DA) release or DA receptor supersensitivity in the mesolimbic pathway may contribute to the positive symptoms of schizophrenia. Problems within the mesocortical pathway, on the other hand, may be connected with negative and cognitive symptoms (Davis et al., 1991). At the same time, the dorsal striatum is exclusively involved in motor function. However, it has recently been suggested that dopaminergic dysfunction in the dorsal striatum and nigrostriatal pathway is most pronounced in schizophrenia (McCutcheon et al., 2019). Positron emission tomography techniques have shown that there is a significant increase of dopamine synthesis and release in the dorsal striatum which is responsible for positive symptoms of schizophrenia development (McCutcheon et al., 2018). Furthermore, robust activity in the dorsal striatum, measured during resting-state fMRI, has been found to correlate with psychotic symptoms (McCutcheon et al., 2019). Thus, it is possible to hypothesize that dopaminergic dysfunction in the dorsal striatum, may contribute to schizophrenia.

In rats with DA transporter knockout, abnormally elevated dopamine level has been shown in the dorsal striatum (Leo et al., 2018). Therefore, it can be hypothesized that the hyperdopaminergic state in DAT-KO rat’s models to some extent may be resemble for certain aspects of the psychotic symptoms of schizophrenia. It was also shown that negative and cognitive symptoms of schizophrenia also depend on norepinephrine transmission (Mäki-Marttunen et al., 2020) and that dopamine and norepinephrine imbalance is involved in the schizophrenia development (Ranjbar-Slamloo and Fazlali, 2020). Our findings demonstrate that stimulating of the norepinephrine release may improve the behavioral tasks acquisition in hyperactive knockout rats with increased dopamine levels in the striatum.

The DAT-KO rats and mice are valuable animal models of ADHD (Gainetdinov et al., 1999; Leo et al., 2018). Mutant rats lacking the dopamine transporter protein DAT, exhibit hyperdopaminergic and motor hyperactivity due to critically high levels of extracellular DA levels in the striatum (Cinque et al., 2018; Leo et al., 2018; Savchenko et al., 2023; Sukhanov et al., 2019). DAT-KO rats and mice are characterized not only by pronounced hyperactivity, but also by a tendency to rigid and stereotyped reactions (Belskaya et al., 2024; Leo et al., 2018; Pogorelov et al., 2005; Sukhanov et al., 2019). Nevertheless, they are able to perform orientation tasks in mazes (Kurzina et al., 2020, 2021), although they are required significantly more time to reach a similar level of learning as WT rats. DAT-KO rats are able to perform spatial and non-spatial behavioral tasks and create their own tactical approaches to obtain rewards (Kurzina et al., 2020, 2021; Savchenko et al., 2022; Volnova et al., 2023). They are most successful in an object recognition task: rats can learn to move an object and retrieve food from the rewarded familiar objects and not to move the non-rewarded novel objects (Kurzina et al., 2021). Interestingly, the knockout animals’ tendency to react stereotypically made them perform this task with fewer errors compared to WT rats, and the learned skill could be retrieved from the memory over the long time, up to 3 months after training (Kurzina et al., 2021). Another characteristic feature of the behavior of DAT-KO rats is their almost complete inability to modify a learned skill due to rigidity and low flexibility in task performance (Belskaya et al., 2024).

In the present work, we have demonstrated how chemogenetic activation of LC neurons can improve the behavioral performance of DAT-KO rats in the Hebb-Williams maze by reducing the number of errors and perseverative reactions. We suppose that parallel functions of DA and NE may facilitate cognitive processes realization. Similar results have previously been obtained in DAT-KO rats following administration of noradrenergic drugs (Kurzina et al., 2022; Volnova et al., 2023).

Acute or repeated administration of the α2A-adrenoceptor agonist guanfacine significantly improved their perseverative activity pattern and reduced the time spent in the maze error zones (Volnova et al., 2023) It is known that guanfacine, the agonist of α2A-adrenoceptor improves numerous PFC functions (Arnsten, 2020). The beneficial effects of guanfacine may arise via strengthening PFC network connectivity as a consequence of NE actions on postsynaptic α2A-adrenoceptors dendrite spines in PFC (Arnsten, 2020; Ota et al., 2021). In contrast, the α2A-adrenoceptor antagonist yohimbine increased the number of perseverative responses (Kurzina et al., 2022). This observation is consistent with the results of chemogenetic suppression of NE transmission from LC neurons to the PFC, which led to the development of perseverative behavior in WT rats (Kabanova et al., 2024a).

In studies using chemogenetic DREADD-induced connectivity, activation of the LC-NE system was found to interrupt ongoing behavior and activate responses to silent stimuli (Zerbi et al., 2019). It has been shown that in working memory tasks, DA is mainly associated with reward expectancy, whereas NE provides memories about the goal and ways to achieve it (Rossetti and Carboni, 2005). The data obtained in our study indicate that increase of NE release from LC to PFC reduced hyperactive behavioral patterns of DAT-KO rats with hyperdopaminergy. A decrease of perseverative activity and visits of erroneous zones were also found. Our previous studies have shown that guanfacine, a selective α2A-adrenoceptor agonist, may improve the fulfillment of spatial working memory tasks in DAT-KO rats (Volnova et al., 2023). Similarly, the present studies show that chemogenetic activation of noradrenergic neurons and norepinephrine release in the prefrontal cortex decreases the number of errors during spatial task performance and the distance traveled in the maze by the DREADD+ group of DAT-KO rats. These results allow us to suggests that stimulation of norepinephrine release may modulate behavioral task learning in hyperactive knockout rats. Our data could form the basis for the development of new, multi-target drugs to treat this pathology.

The potential mechanisms through which the activation of NE release in the PFC may partially compensate the hyperdopaminergia in dopamine transporter knockout rats have not yet been fully elucidated. The mesocortical DA pathways to the PFC originate from the ventral tegmental area, while NE neurons originate in the LC. There is an opinion that dopamine and noradrenaline in PFC modulate higher cognitive functions and are involved in the etiology of pathological states (Ranjbar-Slamloo and Fazlali, 2020). It was proposed that extracellular DA in the cerebral cortex originates not only from dopaminergic terminals but also from noradrenergic ones, where it acts both as precursor for NA as a co-transmitter (Devoto and Flore, 2006). The authors highlight the interaction between NA and DA in the cerebral cortex, suggesting that modulating DA transmission by the noradrenergic system could be an effective drug target for improving cognitive function.

DAT-KO rats, lacking the dopamine transporter, demonstrate multiple alterations in functional connectivity across the brain, particularly prefrontal-midbrain decoupling (Reinwald et al., 2022). The cortico-striatal circuit displayed pronounced hyperconnectivity, whereas thalamic regions and the VTA showed hypoconnectivity with the cortex and striatum. These data allow us to suppose that the NE modulation of DA release in the DAT-KO rat’s PFC may improve its function and optimize behavior.

The findings of this study have to be seen in light of some limitations.

Firstly, viral transduction of target neurons may not ensure that the genetic material reaches all cells of the structure under investigation. Therefore, the effects of chemogenetic stimulation, which do not affect all LC neurons, may not be expressed as strongly at the systemic level (Kabanova et al., 2024b). Our work shows that the efficiency of transduction of noradrenergic LC neurons is significantly lower in DAT-KO than in WT rats. Nevertheless, we have shown that even under these conditions, chemogenetic activation of the LC-to-PFC connections reliably leads to marked changes in expressed behavioral parameters specifically in DAT knockout animals. DAT-KO rats show a decrease in perseverative activity and a reduction in the number of incorrect zones visited in the maze.

Another methodological limitation of our work is the use of animals without the introduced adenoviral vector as a control. We have conducted control behavioral experiments on “DREADD naïve” rats and have demonstrated no effect of Clz at 1 mg/kg dose on distances traveled, duration of test performance, the number of error zone visits, and the number of the return runs. However, some studies have shown a significant effect of clozapine on the distance traveled by control rats microinjected with GFP-expressing AAVs that did not carry genetic information about DREDDs, compared to a saline injection (Gomez et al., 2017). The discrepancy between our findings and those reported by Gomez et al. (2017) may be attributed to the use of different rat strains (Sprague–Dawley rats versus DAT-KO and DAT-WT rats based on the Wistar-Han background). Additionally, the open field locomotor activity tests conducted by Gomez et al. (2017) lasted 30 mintues. In our experiments, by contrast, the rats performed a spatial orientation task in a maze for no more than 1–3 min. Under such brief testing conditions, alterations in locomotor activity have a negligible effect.

In our experiments, the control group of rats (both DAT-KO and WT) did not receive injections of none-DREADD carrying vector. However, we considered it is more important to conduct control experiments confirming the specificity of Clz action as a DREADD ligand. It is particularly significant, because in the literature side effects of clozapine (Clz) and clozapine N-oxide (CNO) that are not related to DREADD activation were revealed. For example, Clz has been shown to affect VTA activity in rat models with induced hyperdopaminergia (Schwieler and Erhardt, 2003). In our experiments using DAT knockout rats exhibiting pronounced hyperdopaminergia, it was essential to rule out any side effects associated with Clz administration at the selected doses. For this purpose, we conducted control behavioral experiments on “DREADD naïve” rats (both DAT-KO and WT) in which had no DREADD expression was present. These experiments demonstrated no effect of Clz on their behavior.

## Conclusion

Thus, the results obtained in this study support an important modulatory role of NE system in hyperactivity and goal-directed spatial cognitive behavior in hyperdopaminergic DAT-KO rats. We may suggest that the interaction between DA and NE plays a leading role in this modulation, and chemogenetic activation of NE neurotransmission in hyperdopaminergic DAT-KO rats can significantly improve the performance of the spatial learning task.

## Data Availability

The raw data supporting the conclusions of this article will be made available by the authors, without undue reservation.
